# Knowledge-enhanced AI drives diagnosis of multiple retinal diseases in fundus fluorescein angiography

**DOI:** 10.3389/fcell.2025.1703606

**Published:** 2025-12-11

**Authors:** Ming-Ming Duan, Hui Qi, Xiang Tu

**Affiliations:** 1 Department of ophthalmology, Jiujiang City Key Laboratory of Cell Therapy, JiuJiang No. 1 People’s Hospital, JiuJiang, Jiangxi, China; 2 Department of Otolaryngology, The Seventh Affiliated Hospital, Sun Yat-sen University, ShenZhen, Guangzhou, China

**Keywords:** artificial intelligence, fundus fluorescein angiography, KeepFIT, retinal diseases, deep learning

## Abstract

**Purpose:**

This study aimed to develop and validate a deep learning model for the accurate multi-class classification of six retinal diseases using fundus fluorescein angiography (FFA) images.

**Methods:**

We applied a knowledge-enhanced pre-training strategy (KeepFIT) using a ResNet-50 image encoder on two large FFA corpora: a curated atlas and a clinical report dataset. The resulting visual encoder was fine-tuned to classify six conditions, including diabetic retinopathy and macular degeneration. The model’s performance and generalizability were assessed on two independent test sets, one of which was sourced from an external institution.

**Results:**

Our proposed deep learning model, leveraging a knowledge-enhanced pre-training strategy, demonstrated robust performance in classifying six distinct retinal diseases using fundus fluorescein angiography images. The model achieved a strong and consistent micro-average area under the curve (AUC) of 0.92 across two independent test sets. Notably, it showed excellent classification performance for critical conditions such as venous occlusion (VO) and neovascular age-related macular degeneration (nAMD), with AUC values reaching 0.95 and 0.96, respectively.

**Conclusion:**

The knowledge-enhanced pre-training strategy significantly improves the diagnostic accuracy and generalizability of deep learning models for FFA analysis. This approach provides a scalable and effective framework for automated retinal disease screening, holding significant potential for clinical decision support, especially in resource-limited settings.

## Introduction

With the aging of the global population, retinal diseases, including diabetic retinopathy and age-related macular degeneration, are increasing worldwide and are increasingly becoming a significant cause of eye health hazards, leading to visual impairment and even blindness (Burton et al., 2021). Early screening and timely intervention can help prevent or minimize eye damage caused by retinal diseases (Vujosevic et al., 2020). However, large-scale manual screening is difficult to achieve due to limited medical resources. Therefore, accurate, cost-effective, and efficient screening methods for retinal diseases are essential. Fundus fluorescein angiography (FFA) is an important ophthalmic imaging technique. The technique uses sodium fluorescein as a contrast agent injected intravenously, and its fluorescent properties allow details of the retinal blood vessels to be visualized when the fundus is illuminated with specific wavelengths (Wang et al., 2017; Liu et al., 2025). Thus, FFA can assess microvascular structure and blood flow and is the gold standard for the diagnosis of fundus diseases such as diabetic retinopathy (Yu et al., 2025). However, the interpretation of FFA images is time-consuming, and the diagnosis is dependent on the ophthalmologist’s expertise and subjective, which makes the efficiency and accuracy of the diagnosis, to some extent, compromised (Gao et al., 2023).

In recent years, the rapid development of artificial intelligence (AI) technology has brought new possibilities for the screening and diagnosis of fundus diseases. Deep learning-based diagnostic systems have been widely used in the detection of various fundus diseases, such as diabetic retinopathy and age-related macular degeneration (Long et al., 2025; Dai et al., 2021; Dai et al., 2024; Yim et al., 2020; Kazemzadeh, 2025), showing great potential for clinical decision support. It can quickly and accurately identify and analyze the characteristics of fundus lesions, which greatly improves the efficiency and accuracy of early screening and diagnosis of various fundus diseases. However, many previous studies have typically utilized separate datasets to train task-specific models, which leads to poor generalization across different scenarios and the need for large amounts of annotated training data (Yang et al., 2022). MM-Retinal is a multimodal dataset that includes graphic paired data from FFA, color fundus photography (CFP), and optical coherence tomography (OCT) images (Wu R. et al., 2024). The data were obtained from fundus chart books containing comprehensive ophthalmologic knowledge and accurate graphic descriptions provided by ophthalmologists. The knowledge-enhanced foundational pre-training model which incorporates fundus image-text expertise, known as KeepFIT, is a knowledge-enhanced base model that injects MM-Retinal’s fundus expert knowledge into model training through an image similarity-guided text modification approach and a hybrid training strategy (Wu R. et al., 2024). It exhibits strong robustness and generalizability.

Therefore, this study uses the KeepFIT model introduced with MM-Retinal for pre-training, with ResNet-50 as the image encoder $E_{V}$ and BioClinical-BERT as the text encoder $E_{V}$ . The domain knowledge of MM-Retinal–FFA is injected into the labeled cue text of fluorescein fundus angiography image repository (FFA-IR) via an image similarity-based text correction module. The trained visual backbone $E_{V}$ was then transferred to the classification task to classify the FFA dataset containing central serous chorioretinopathy (CSC), venous occlusion (VO), neovascular age-related macular degeneration (nAMD), non-proliferative diabetic retinopathy (NPDR), proliferative diabetic retinopathy (PDR), and healthy control. The aim of this study was to improve the efficiency and accuracy of diagnosis of fundus diseases.

## Methods

### Pre-training data

The foundation model is first exposed to two complementary vision–language corpora that both belong to the FFA modality. The first source is MM-Retinal–FFA, a subset of the MM-Retinal atlas that offers 1,947 high-resolution (≥800 × 800) FFA photographs, each accompanied by a bilingual expert description. The second source is FFA-IR, a clinical collection composed of 1,048,584 FFA frames aligned with 10,790 bilingual diagnostic reports that follow a 46-label schema. Because FFA-IR is two orders of magnitude larger than MM-Retinal–FFA, every mini-batch is constructed with a one-to-one ratio of samples from the two corpora to maintain a balance between scale and expert coverage.

### Downstream classification data

**FIGURE 1:**
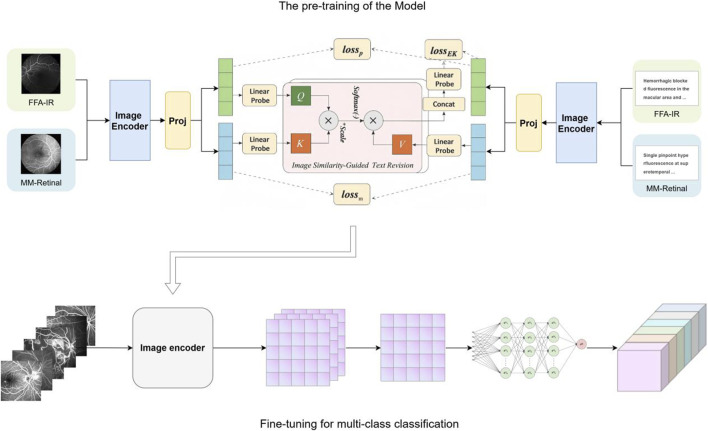
Flowchart of the experiment.

In this retrospective study, we sourced a primary dataset of FFA images from patients treated at JiuJiang No. 1 People’s Hospital between January 2017 and December 2022. This dataset was randomly divided, with 80% of the images allocated for training and 20% for validation. Additionally, an independent internal test set (Test Set 1) was constructed using images acquired at the same hospital between January 2023 and January 2025. To further assess generalizability, an external test set (Test Set 2) was compiled using FFA images collected during the same period from Seventh Affiliated Hospital, Sun Yat-sen University. The research was conducted in accordance with the tenets of the Declaration of Helsinki and received approval from the institutional review boards of both hospitals. Given that the study relied exclusively on anonymized data devoid of personal identifiers, the ethics committees granted a waiver for patient-specific informed consent. The overall study framework is illustrated in Figure 1.

### Knowledge-enhanced vision–language pre-training

Pre-training follows the KeepFIT strategy that was introduced together with MM-Retinal. A ResNet-50 model initialized on ImageNet-1 K serves as the image encoder $E_{V}$ ; BioClinical-BERT acts as the text encoder $E_{t}$ . In addition, the standard ResNet-50 model from the Torchvision library serves as the image encoder without any architectural modification; BioClinical-BERT acts as the text encoder. Another domain-specific language model based on BERT, pre-trained on large-scale clinical notes, is included, enabling it to effectively understand medical terminology and context (Lee et al., 2020). Their outputs are projected into a shared 512-dimensional space through linear layers with layer normalization. To inject domain knowledge from MM-Retinal–FFA into the label-prompt texts of FFA-IR, an image similarity-guided text-revision module is implemented as an eight-head cross-attention block.

Let B be a mini-batch that mixes the two datasets. Genuine image–caption pairs from MM-Retinal–FFA are trained with a symmetric InfoNCE contrastive loss Lm. Prompt pairs from FFA-IR are optimized by a category-aware variant $L_{P}$ that attracts images whose prompts share the same disease label. The revised text embedding $E_{K}$ produced by the cross-attention module is encouraged to remain close to the original prompt embedding $t_{p}$ via a mean-squared error term $L_{EK}$ . The complete objective is given as

$$L=Lm+Lp+\alpha L_{EK},\alpha =100.$$

Optimization uses AdamW, with an initial learning rate of $1\;X\;10^{‐4}$ and weight decay of $1\;X\;10^{‐5}$ . Training runs for 20 epochs on four RTX 4090 GPUs, with a per-GPU batch size of 64.

### Fine-tuning for multi-class classification

The pre-trained visual backbone $E_{V}$ is transferred to the classification task. All layers are unfrozen. The final convolutional feature map is transformed by global average pooling into a 2048-dimensional vector that feeds a four-layer multilayer perceptron. Cross-entropy is minimized with Adam (a learning rate of $1\;X\;10^{‐4}$ and a weight decay of $1\;X\;10^{‐5}$ ). The learning rate decreases by a factor of 10 every 10 epochs; training terminates after 10 epochs. Data augmentation at training time includes random horizontal flip, ±10° rotation, and mild color jitter. At inference time, the softmax of the six logits yields posterior probabilities for each diagnosis.

## Results

### Characteristics of the datasets

**FIGURE 2:**
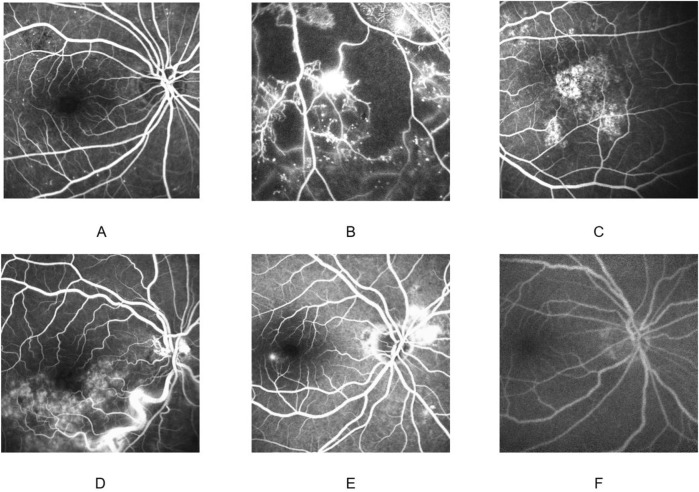
Fundus fluorescein angiography findings in different diseases. Non-proliferative diabetic retinopathy **(A)**; proliferative diabetic retinopathy **(B)**; neovascular age-related macular degeneration **(C)**; venous occlusion **(D)**; central serous chorioretinopathy **(E)**; normal **(F)**.

Fine-tuning and evaluation are conducted on an independent FFA dataset that covers six clinically important categories: CSC, VO, nAMD, NPDR, PDR, and healthy control. The training set contains 5,270 images (CSC 790, VO1, 650, nAMD 1,300, NPDR 692, PDR 429, and normal 409). The validation set comprises 1,054 images (CSC 300, VO 300, nAMD 300, NPDR 75, PDR 40, and normal 39). Two disjoint test sets are used: Test Set 1 with 1,908 images (CSC 500, VO 500, nAMD 400, NPDR 215, PDR 166, and normal 127) and Test Set 2 with 896 images (CSC 300, VO 300, nAMD 120, NPDR 98, PDR 55, and normal 23). All frames are resized to 224 × 224 pixels, center-cropped if necessary, and normalized using the ImageNet mean and standard deviation (mean = [0.485, 0.456, and 0.406]; std = [0.229, 0.224, and 0.225]). The distribution of images of each retinal disease category in the training set, validation set, and test sets is detailed in Table 1. Representative examples of each disease category are illustrated in Figure 2.

**TABLE 1:** Dataset characteristics.

Category	Training set	Validation set	Test set 1	Test set 2
CSC	790	300	500	300
VO	1,650	300	500	300
nAMD	1,300	300	400	120
NPDR	692	75	215	98
PDR	429	40	166	55
Normal	409	39	127	23

CSC, central serous chorioretinopathy; VO, venous occlusion; nAMD, neovascular age-related macular degeneration; NPDR, non-proliferative diabetic retinopathy; PDR, proliferative diabetic retinopathy.

### Evaluation of model diagnostic performance

The deep learning model developed in this study demonstrated favorable multi-class classification performance across six retinal disease categories in both internal and external test sets. The overall accuracy ranged from 0.868 to 0.967, and the micro-average AUC remained consistently high at 0.92 in both datasets. Nevertheless, classification performance varied across disease types, with notable differences observed in precision, sensitivity, and F1-scores.

The model achieved the most consistent and robust performance in identifying central serous chorioretinopathy and retinal vein occlusion. In Test Set 1, the F1-scores for these two categories reached 0.746 and 0.795, respectively, and further improved to 0.788 and 0.818, respectively, in Test Set 2. Both precision and sensitivity remained high, indicating a well-balanced classification capability.

These findings were further supported by the receiver operating characteristic analysis. The AUCs for central serous chorioretinopathy were 0.91 and 0.92 in the two test sets, while those for retinal vein occlusion were 0.89 and 0.90, respectively. The micro-average AUC of 0.92 in both test sets reflects the model’s overall stable classification performance.

**FIGURE 3:**
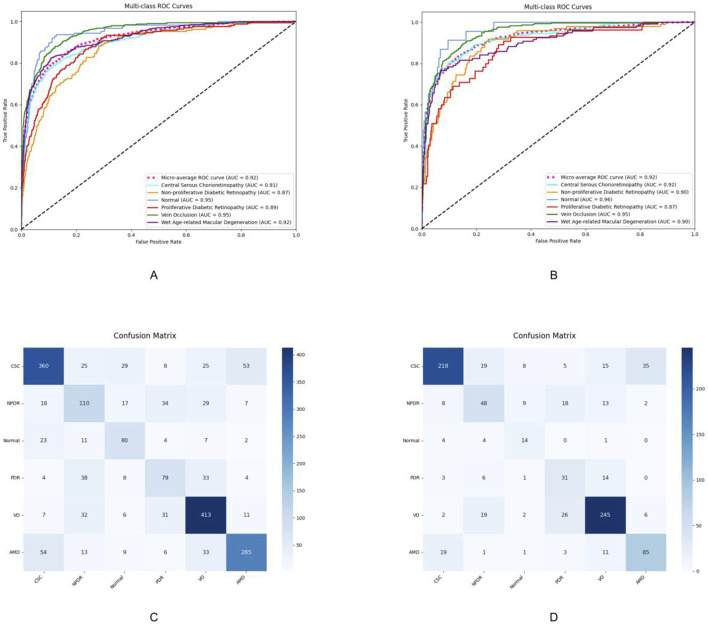
Performance evaluation results of the model on Test Set 1 and Test Set 2. **(A,C)** ROC curves and confusion matrices of the model on Test Set 1, respectively. **(B,D)** ROC curves and confusion matrices of the model on Test Set 2, respectively. CSC, central serous chorioretinopathy; VO, venous occlusion; nAMD, neovascular age-related macular degeneration; NPDR, non-proliferative diabetic retinopathy; PDR, proliferative diabetic retinopathy.

Confusion matrix analysis also revealed that central serous chorioretinopathy and retinal vein occlusion had the highest numbers of correctly classified cases, with 360 and 218 true positives for central serous chorioretinopathy and 413 and 245 for retinal vein occlusion in the two test sets, respectively. Misclassification rates for these categories were comparatively low, underscoring the model’s strong and generalizable discriminatory capacity. The detailed diagnostic performance metrics are summarized in Table 2, and the corresponding ROC curves and confusion matrices are presented in Figure 3.

**TABLE 2:** Model performance evaluation on the validation and test sets.

Task	Accuracy	Precision	Sensitivity	Specificity	F1-score
Test set 1					
CSC	0.871	0.772	0.720	0.925	0.746
PDR	0.883	0.481	0.512	0.930	0.497
Normal	0.940	0.537	0.630	0.962	0.580
NPDR	0.911	0.488	0.476	0.952	0.481
VO	0.888	0.765	0.826	0.910	0.795
nAMD	0.900	0.787	0.713	0.949	0.748
Test set 2					
CSC	0.868	0.858	0.727	0.940	0.788
PDR	0.890	0.495	0.490	0.939	0.492
Normal	0.967	0.400	0.609	0.976	0.483
NPDR	0.915	0.373	0.564	0.939	0.449
VO	0.878	0.819	0.817	0.909	0.818
nAMD	0.913	0.664	0.708	0.945	0.685

CSC, central serous chorioretinopathy; VO, venous occlusion; nAMD, neovascular age-related macular degeneration; NPDR, non-proliferative diabetic retinopathy; PDR, proliferative diabetic retinopathy.

## Discussion

In this study, we developed and validated a deep learning model for the classification of six common retinal diseases using FFA images. Our primary finding is that a visual model pre-trained with KeepFIT demonstrates both high accuracy and robust generalizability. The model achieved a consistent micro-average AUC of 0.92 across two independent test sets, one of which was sourced from an external institution, underscoring its potential for broader clinical application and affirming our initial hypothesis.

Our model’s capacity for simultaneous differential diagnosis among six distinct retinal conditions marks a significant advancement over prior work, which has predominantly focused on binary classification tasks (Lee et al., 2021; Heydon et al., 2021; Alsaih et al., 2017; N et al., 2021). Although previous models have achieved high performance in detecting single, specific lesions such as neovascular leakage in PDR (Yang et al., 2022), their utility is confined to narrow clinical questions. In contrast, our model’s ability to differentiate between multiple pathologies, including CSC, VO, nAMD, and different stages of DR (Long et al., 2025; Gan et al., 2023), more closely mirrors the complex diagnostic challenges encountered in real-world clinical practice (Shao et al., 2025; Ueno et al., 2019). This multi-class capability significantly broadens its potential applicability as a decision support tool for ophthalmologists.

The key innovation of our study lies in the KeepFIT pre-training methodology, which distinguishes our approach from conventional supervised learning paradigms used in earlier multi-class FFA classifiers (Gao et al., 2023; Huang et al., 2024). Traditional models rely heavily on learning pixel-level patterns from labeled images, a process that is often constrained by the quantity and quality of available annotations, as labels can be highly subjective and exhibit large variance between experts (Wu J. et al., 2024; Chen, 2023; Hu et al., 2021). Our strategy fundamentally circumvents this limitation by leveraging two heterogeneous data sources: a curated atlas dataset providing precise, granular knowledge and a large-scale clinical report dataset capturing the rich, nuanced language of expert interpretation. This allows the model to move beyond superficial feature recognition and learn the deep semantic correlations between imaging findings and their underlying pathology. Consequently, the model acquires a foundational “understanding” of retinal diseases, which enhances its generalization capabilities and reduces its dependency on massive, meticulously annotated datasets for fine-tuning.

The model’s performance, while strong overall, varied across different disease categories. It demonstrated exceptional classification accuracy for nAMD and VO, with AUC values reaching as high as 0.96 and 0.95, respectively. This success can likely be attributed to the distinct and often dramatic morphological changes these conditions present on FFA imaging, such as neovascular leakage or clear vascular blockages, providing strong signals for the model to learn from. Conversely, the F1-scores for NPDR and PDR were comparatively lower. This disparity may stem from two factors. First, the training dataset contained fewer examples of PDR and NPDR compared to VO and nAMD, creating a data imbalance that could impede learning, a common challenge in medical AI development (Gan et al., 2025a; Gan et al., 2025b; Li et al., 2021). Second, the pathological features of early diabetic retinopathy (Gan et al., 2025b) can be subtle and varied, posing a greater challenge for automated detection compared to the more overt features of other diseases.

However, we acknowledge several limitations. First, the aforementioned class imbalance in the fine-tuning dataset remains a significant constraint. Future efforts should prioritize the curation of larger and more balanced multi-class datasets to further refine model performance, particularly for underrepresented diseases. Second, although validated on data from two centers, establishing true global generalizability requires further testing on more geographically and ethnically diverse multinational cohorts as models trained on data from a single center or limited population may lack generalizability (Gan et al., 2023; Gong et al., 2024). Third, the “black box” nature of the current model is a common barrier to clinical translation. Integrating established interpretability techniques, such as class activation maps (CAMs), is a critical next step (Wang et al., 2020). This will not only enhance transparency by providing visual evidence for the model’s predictions but also foster greater trust and adoption among clinicians who need to understand the basis of the AI’s diagnostic reasoning.

## Conclusion

This study demonstrates that a knowledge-enhanced vision–language pre-training strategy significantly improves the diagnostic accuracy and generalizability of deep learning models for fundus fluorescein angiography image classification. By addressing the challenge of efficient and scalable retinal disease screening, the proposed approach meets the critical need for intelligent diagnostic tools in ophthalmology. These findings advance the integration of clinical expertise into medical AI and offer a promising framework for real-world application in resource-limited settings.

## Data Availability

The raw data supporting the conclusions of this article will be made available by the authors, without undue reservation.
